# Items

**Published:** 1901-12

**Authors:** 


### ITEMS.

Prof. H. Schweitzer, formerly professor of chemistry in the University of Heidelburg, recently visited Detroit in relation to a legal controversy growing out of counterfeiting the trade-marks owned by Farbenfabriken of Elberfeld Co. charged against some men in Detroit. During his visit Prof. Schweitzer visited the establishment of Parke, Davis & Co., and according to the *Detroit Journal* remarked that it is the greatest industry of the kind in the world, the greatest beyond all question. The biological department is astounding. The physical assay work on animals is worth to a student a walk of 1,000 miles. The scientific atmosphere is an inspiration and the ingenious machinery a marvel. I was told that there were employed in the factory alone over 1,500 people, and that the firm has 207 traveling men employed.

There are five American branches, I was told, and there are manufacturing plants in England and Canada. In the English plant are employed 250 persons. There is nothing wanting in this plant for the production of powerful, accurate, uniform and palatable medication. They have a circulating library for the employees, as well as an emergency hospital, and I understand the employees have decent hours and are well treated.

A Handsome Group of Portraits.—About a year ago, within the short period of three weeks, four eminent and distinguished physicians departed this life. Two of these well known men, Hunter McGuire and Lewis A. Sayre, were surgeons and two, Jacob M. DaCosta and Alfred L. Stillé, physicians, and three of them were former presidents of the American Medical Association. Believing that the rank and file of the profession would appreciate and preserve portraits of these representative practitioners and teachers, the Arlington Chemical Company commissioned a competent artist to paint them in oil in the shape of a panel suitable for framing, and have reproduced the painting for distribution to their friends in the profession. This handsome and thoroughly worthy group of portraits is now being mailed and if any physician is, perchance, omitted from the list, a request will bring a copy.

